# The impact of lenvatinib on sarcopenia in patients with advanced unresectable hepatocellular carcinoma

**DOI:** 10.1038/s41598-024-66766-8

**Published:** 2024-09-27

**Authors:** Michael Praktiknjo, Ana S. Pena Solano, Farsaneh Sadeghlar, Thomas Welchowski, Matthias Schmid, Christian Möhring, Taotao Zhou, Robert Mahn, Malte B. Monin, Carsten Meyer, Georg Feldmann, Peter Brossart, Cornelius van Beekum, Alexander Semaan, Hanno Matthaei, Steffen Manekeller, Alois M. Sprinkart, Sebastian Nowak, Julian Luetkens, Jörg C. Kalff, Christian P. Strassburg, Maria A. González-Carmona

**Affiliations:** 1 Department of Internal Medicine I, University Hospital Bonn, Bonn, Germany; 2 Department of Internal Medicine B, University Hospital Münster, Münster, Germany; 3 Department of Medical Biometry, Informatics and Epidemiology (IMBIE), University Hospital Bonn, Bonn, Germany; 4 Department of Diagnostic and Interventional Radiology, University Hospital Bonn, Bonn, Germany; 5 Department of Internal Medicine III, University Hospital Bonn, Bonn, Germany; 6 Department of Visceral Surgery, University Hospital Bonn, Bonn, Germany; 7 Department of General- Abdominal and Transplant Surgery, Hanover Medical School, Hannover, Germany

**Keywords:** Hepatocellular carcinoma, Chemotherapy, Cancer, Immunology, Oncology

## Abstract

Lenvatinib is a multiple receptor tyrosine kinase inhibitor (TKI) approved for first-line treatment of patients with unresectable hepatocellular carcinoma (HCC). TKI are suspected of exacerbating muscle loss in patients with cancer. In this study, we analyze the role of muscle loss in patients with advanced HCC treated with lenvatinib. This is a retrospective analysis of a real-life cohort of 25 patients with advanced HCC who were treated with lenvatinib from 2018 to March 2021 in Germany. Patients were stratified for loss of skeletal muscle area during the first three months of lenvatinib therapy. Overall survival (OS), progression-free survival (PFS) and toxicity were analyzed for all patients, especially regarding loss of muscle before and during the first three months of therapy with lenvatinib. Three months after beginning of therapy with lenvatinib, a significant reduction of muscle mass was observed in 60% of patients (*p* = 0.035). Despite increase of loss of skeletal muscle, patients benefitted from lenvatinib in our cohort of patients in terms of OS and PFS and did not experience increased toxicity. Furthermore, muscle loss was not a negative predictor of survival in the univariate analysis (*p* = 0.675). Patients with advanced hepatocellular carcinoma experience muscle loss with lenvatinib therapy. However, despite progressive muscle loss, patients benefit from a therapy with lenvatinib in terms of OS and PFS without increased toxicity. However, assessment and prophylaxis of skeletal muscle status should be recommended during a therapy with lenvatinib.

## Introduction

Hepatocellular carcinoma (HCC) is the most common primary liver malignancy and the third leading cause of cancer-related death worldwide. Despite recent advances in the systemic treatment of advanced HCC, the prognosis of HCC is still poor. Chronic viral hepatitis B/C, alcohol use disorder, metabolic liver disease (particularly nonalcoholic fatty liver disease) and aflatoxins are the most frequent risk factors for HCC. The molecular mechanisms of HCC progression have not yet been completely clarified^1–3^.

The relevant parameters for determining prognosis and therapy of HCC are defined by the Barcelona Classification for Liver Cancer (BCLC). Further prognostic scores or parameters, such as ALBI, but also ECOG status and clinical symptoms, may be applied to predict HCC prognosis. HCC is often diagnosed at advanced stages (BCLC C). In accordance with international guidelines such as those of EASL or ESMO, it is recommended to start first-line systemic therapy with atezolizumab (a PD-L1 antibody) in combination with bevacizumab or with tremelimumab (a CTLA-4 antibody) in combination with durvalumab (a PD-L1 antibody). This recommendation is supported by data from the phase III IMbrave150 trial and the Himalaya study. In case of toxicity or contraindications, oral therapy with sorafenib or lenvatinib is recommended^4–6^.

Lenvatinib was approved in 2018 for the treatment of advanced HCC as first-line therapy. Based on results from the randomized phase III REFLECT trial, lenvatinib was not inferior to sorafenib regarding overall survival (OS)^7^. Side effects related to lenvatinib were manageable with dose modifications. In addition, it has been reported that high serum lenvatinib levels resulted in weight (muscle and fat) loss^8^. However, weight loss did not serve as a good predictor of prognosis to lenvatinib therapy in contrast to muscle loss^7,9^.

Sarcopenia defined as loss of skeletal muscle mass and function can easily be measured using computed tomography (CT) or magnetic resonance imaging (MRI)^10^. We and others have shown that sarcopenia is common in patients with advanced liver disease or malignancies and that it represents a relevant independent prognostic factor in these patients^11–17^.

For instance, measurement of muscle mass at the third lumbar vertebra (L3) using CT has been shown to be a strong independent predictor of mortality in patients with cirrhosis or HCC^18–20^. Furthermore, muscle depletion can lead to physical disability in HCC patients, resulting in reduced tolerability to chemotherapy and TKI^20^. Finally, it has been shown that several TKIs can aggravate patients' sarcopenia due to different mechanisms. The presence of presarcopenia or sarcopenia before administration of lenvatinib has been identified as a significant prognostic factor and a predictive marker for tolerability to lenvatinib in patients with advanced HCC and treated with lenvatinib in several studies published to date. However, these studies focused on the impact of sarcopenia before therapy with lenvatinib on outcome and tolerability to lenvatinib and not on the impact of lenvatinib on sarcopenia during treatment. Noteworthy, existing studies predominantly originate from Asia, particularly Japan and China (Table 1)^21–26^.

**Table 1:** Baseline and therapy characteristics.

	All patients n = 25 (%)	Non muscle loss during 3 months lenvatinib n = 6 (%)*	Muscle loss during 3 months lenvatinib n = 15 (%)*	*p* value
Age				1
< 65	12 (48%)	3 (50%)	8 (53.2%)	
≥ 65	13 (52%)	3 (50%)	7 (46.6%)	
Sex				1
Male	16 (64%)	4 (66.7%)	10 (66.7%)	
Female	9 (36%)	2 (33.3%)	5 (33.3%)	
ECOG				0.9544
0–1	19 (76%)	5 (83.3%)	13 (86.7%)	
≥ 2	6 (24%)	1 (16.7%)	2 (13.3%)	
Etiology				0.91123
HBV	5 (20%)	2 (33.3%)	2 (13.3%)	3
HCV	3 (12%)	1 (16.7)	2 (13.3%)	
Alcohol	6 (24%)	1 (16.7%)	3 (20%)	
NASH/NAFLD	6 (24%)	1 (16.7%)	4 (26.7%)	
Other/unknown	5 (20%)	1 (16.7%)	4 (26.7%)	
Child Pugh score				0.54355
A	20 (80%)	4 (66.6%)	13 (86.5%)	8
B	5 (20%)	2 (33.3%)	2 (13.3%)	
MELD > 6 Points	21 (84%)	5 (83.3%)	12 (79.9%)	1
BCLC C	25 (100%)	6 (100%)	15 (100%)	1
Macroscopic MVP infiltration	12 (48%)	4 (66.6%)	4 (26.6%)	1
Extrahepatic metastasis	19 (76%)	6 (100%)	9 (59.9%)	1
AFP (ng/ml)				0.63461
< 200	13 (52%)	4 (66.6%)	7 (46.6%)	7
≥ 200	12 (48%)	2 (33.3%)	8 (53.2%)	
ALBI				0.44657
Grade 1	8 (36%)	3 (49.9%)	4 (26.6%)	
Grade 2	14 (56%)	3 (49.9%)	8 (53.2%)	9
Grade 3	3 (12%)	0 (0%)	3 (19.9%)	
BMI				1
≤ 18.5	3 (12%)	1 (16.7%)	1 (6.6%)	
> 18.5 to ≤ 25	8 (20%)	2 (33.3%)	6 (39.9%)	
> 25	14 (72%)	3 (50%)	8 (53.2%)	
Weight (kg)				1
≤ 60	6 (24%)	1 (16.7%)	4 (26.6%)	
> 60	19 (76%)	5 (83.3%)	11 (73.3%)	
Muscle mass assesment at beginning/during 3 months lenvatinib therapy				0.36130
Non-sarcopenia	14 (56%)	2 (13.3%)	9 (59.9%)	3
Sarcopenia	11 (44%)	4 (26.6%)	6 (40%)	
Previous therapy				0.873678
Liver transplantation	2 (8%)	0 (0%)	2 (13.3%)	
RFA	4 (16%)	1 (16.7%)	3 (20%)	
Resection	12 (48%)	6 (100%)	4 (26.6%)	
TACE	11 (44%)	4 (66.7%)	6 (40%)	
SIRT	5 (20%)	2 (33.3%)	2 (13.3%)	
Radiation	1 (4%)	1 (16.6%)	0 (0%)	
Sorafenib	5 (20%)	1 (16.7%)	3 (20%)	
Regorafenib	1 (4%)	0 (0%)	1 (6.7%)	
PD-1 antibody	2 (8%)	1(16.7%)	1 (6.7%)	
Ramucimumab	1 (4%)	0 (0%)	1 (6.7%)	
Cabozantinib	2 (8%)	1 (16.7%)	1 (6.7%)	
Start dosis lenvatinib (mg/d)				0.443842
4	9 (36%)	1 (16.6%)	5 (33.3%)	
8	13 (52%)	5 (83.3%)	7 (46.7%)	
12	3 (12%)	0 (0%)	3 (20%)	
Lenvatinib				1
First line	20 (80%)	5 (83.3%)	12 (80%)	
≥ Second line	5 (20%)	1 (16.7%)	3 (20%)	
Therapy after lenvatinib				0.061203
Sorafenib	6 (24%)	0 (0%)	6 (40%)	
Pembrolizumab	1 (4%)	1 (16.7%)	0 (0%)	
Pembrolizumab + Lenvatinib	1 (4%)	1 (16.7%)	0 (0%)	
Ramucimumab	2 (8%)	1 (16.7%)	1 (6.7%)	
Cabozantinib	1 (4%)	0 (0%)	1 (6.7%)	

Baseline characteristics of patients at the beginning of lenvatinib and three months after lenvatinib therapy.

Numbers are presented as n (%).

*ECOG* Eastern Cooperative Oncology Group performance status; *BCLC* Barcelona Clinic Liver Cancer; *AFP* alpha-fetoprotein; *BMI* body mass index; *ALBI* albumin-bilirubin score; *MVP* main portal vein; *NASH* non-alcoholic steatohepatitis; *NAFLD* non-alcoholic fatty liver disease; *HBV* hepatitis B virus; *HCV* hepatitis C virus; *SIRT* selective internal radiotherapy; *TACE* transarterial chemoembolization; *MELD* model of end stage liver disease; *PD-1* programmed cell death protein 1.

*Only 21 patients were evaluated, since four patients died before first CT-staging.

In the study presented by Uojima et al.^24^ decreased muscle mass was associated with increased severe AEs and reduced OS. Another substantial retrospective trial presented by Hiraoka et al.^21^ in 2021, comprising 151 patients from Japan, presarcopenia before onset of therapy with lenvatinib emerged as a significant prognostic factor for survival.

Regrettably, comprehensive data analyzing skeletal muscle development during lenvatinib therapy in advanced HCC from a European cohort are scarce. A sole report from Europe, inclusive of two cases from the REFLECT study in Italy, has been published to date. In this study, muscle mass loss was tracked over 24 months, with the observed overall survival (34 and 42 months) and duration of lenvatinib therapy (25 and 32 months) exceeding those reported in other cases^22^.

In the retrospective analysis by Endo et al.^23^, 63 patients with advanced HCC and treated with lenvatinib were analyzed. A decreased grip strength (GS) and decreased skeletal muscle mass index (SMI) were found in 33.3% and 34.9% of the patients, respectively. In this study, only GS seemed to have an impact on survival, since OS of the normal GS group was significantly higher than of the decreased GS group, while that of the normal and decreased SMI groups did not significantly differ, indicating that muscle strength and not just muscle mass alone may be also relevant as a prognostic marker^23^.

For example, in the study presented by Uojima et al.^24^ in 2020, involving 100 patients from Japan, decreased muscle mass correlated with increased severe AEs and reduced OS. Muscle mass assessment was conducted solely before lenvatinib therapy and defined as skeletal muscle index (SMI).

The available data is limited and controversial. While some studies show the effects of lenvatinib on muscle mass, other studies are inconclusive. A subanalysis of the prospective SORAMIC trial does not suggest a significant impact of sarcopenia on the survival of patients with advanced HCC. Consequently, sarcopenia does not appear to play a substantial role in patient allocation within this palliative treatment cohort^27^. It‘s important to note that these studies lacked a longitudinal analysis of the effects of lenvatinib on sarcopenia.

Therefore, the objective of this study was to examine the impact of lenvatinib on muscle mass during treatment in a European cohort of patients with advanced HCC.

## Results

### Base line characteristics

Twenty-five patients treated with lenvatinib were enrolled in the study between June 2018 and March 2021. The median observation period was seven months (range = 0–23 months). Base line characteristics of all patients are presented in Table 2. Sixteen of the patients were male (64%) and median age was 67 (39–81 years). At the start of lenvatinib therapy, 76% (19) of the patients presented an Eastern Cooperative Oncology Group (ECOG) performance status of 0 or 1 (eight patients ECOG 0, 11 patients ECOG 1). All patients had advanced HCC corresponding to BCLC stage C, of whom 12 (48%) patients presented with macroscopic infiltration of the main portal vein and 19 patients (76%) with extrahepatic metastasis. In 48% (12) AFP level was ≥ 200 ng/ml. The ALBI score was 1 in 36% (8), 2 in 14 patients (56%) and 3 in three patients (12%). Regarding comorbidities, 80% (20) had concomitant Child–Pugh A liver cirrhosis and 20% (5) Child–Pugh B liver cirrhosis (four patients with 7 points, one patient with 8 points). Among the causes of liver cirrhosis, the following were the most common: chronic hepatitis B 20% (5), chronic hepatitis C 12% (3), alcohol 24% (6) and NASH/NAFLD 24% (6). Regarding treatment characteristics, nine (36%) patients received 4 mg/day as starting dose of lenvatinib and 52% (13) received 8 mg/day*.* Only 12% (3) received a 100% lenvatinib dose of 12 mg/day as starting dose. Interestingly, five patients (20%) received lenvatinib as second-line treatment, 8% (2), 4% (1) as third-line, and 4% (1) as fourth- line and even fifth-line therapy. Further previous and follow-up therapies are documented in Table 2. Regarding weight and muscle mass, 76% (19) of the patients weighed above 60 kg and 72% (14) were overweight with a BMI > 25. Interestingly, from the diagnosis of HCC to the start of lenvatinib therapy, 52% (12) had already lost muscle mass.

**Table 2:** Objective response rate and disease control rate.

	All patients n = 25 (%)	Non muscle loss during 3 months lenvatinib n = 6 (%)*	Muscle loss during 3 months lenvatinib n = 15 (%)*
PD	11 (44%)	1 (16.7%)	8 (53.3%)
SD	5 (20%)	3 (50%)	1 (6.7%)
PR	4 (16%)	1 (16.7%)	3 (20%)
CR	0 (0%)	0 (0%)	0 (0%)
Unknown	5 (20%)	1 (16.7%)	3 (20%)
ORR	4 (16%)	1 (16.7%)	3 (20%)
DCR	9 (36%)	4 (66.7%)	4 (26.7%)

Numbers are presented as n (%).

*HCC* hepatocellular carcinoma; *CR* complete response; *PR* partial response; *SD* stable disease; *PD* progressive disease; *ORR* objective response rate; *DCR* disease control rate.

*Only 21 patients were evaluated, since four patients died before first CT staging.

### Muscle development during lenvatinib therapy

**Figure 1:**
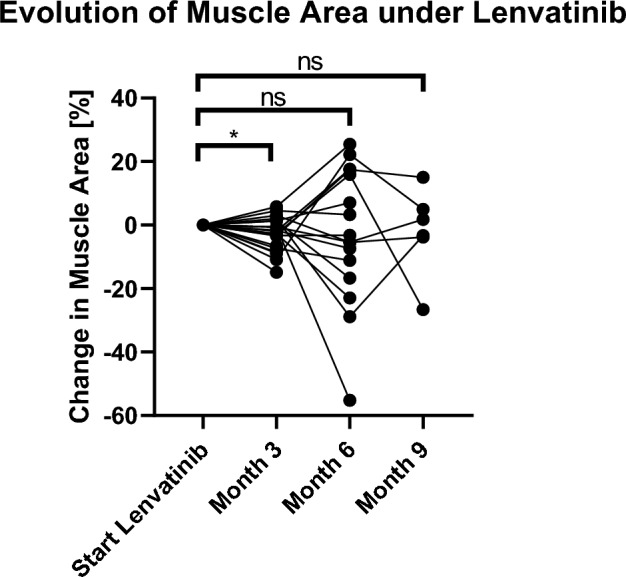
Three months after starting of lenvatinib therapy, development of muscle mass of 21 patients could be evaluated. Of these, 15 showed muscle atrophy (*p* = 0.035). A significant decrease of muscle loss (− 6.5% ± 3.3%; *p* = 0.035) in the first three months of lenvatinib therap in the majority of patients (60%) was detected.

For the present study, 150 CT imaging data were evaluated at different time-points as described in the methods section. Three months after onset of therapy with lenvatinib, development of muscle mass in 21 patients could be evaluated. Four patients died before first CT staging and had to be ruled out from analysis. Of the remaining 21 patients, 15 patients lost muscle mass during the first three months of treatment with lenvatinib (*p* = 0.035) as shown in Fig. 1. Interestingly, in our cohort, patients developing muscle loss during the first three months of lenvatinib intake (n = 15) showed similar baseline and therapy characteristics when compared to patients without muscle loss (n = 6) (Table 2). 2 representative CT images for measuring the SMA at the start and 3 months after the start of lenvatinib therapy can be found as supplementary Figure S1. In particular, no differences were detected in performance status according to ECOG score (*p* = 0.9544). Of note, regarding liver function, 33.3% (2) in the non-muscle loss group presented with worse liver function at onset of lenvatinib therapy versus two patients (13.3%) in the muscle loss group (*p* = 0.5436).

In the non-muscle loss group, no patients with Albi grade 3 were observed, while in the muscle loss group, 19.9% (3) featured an ALBI grade 3 (*p* = 0.4466).

The remaining parameters (e.g. age, sex, etiology of HCC or previous treatments) were similar between the two patient groups. Mainly, there was no difference between BMI and muscle mass status during the three months of lenvatinib therapy (Table 2).

Additionally, the calculation of the fat-free muscle fraction did not show significant differences between the groups with muscle loss and those without muscle loss (Table S2).

### Efficacy of lenvatinib in all patients and influence of muscle loss on OS, PFS and ORR

**Figure 2:**
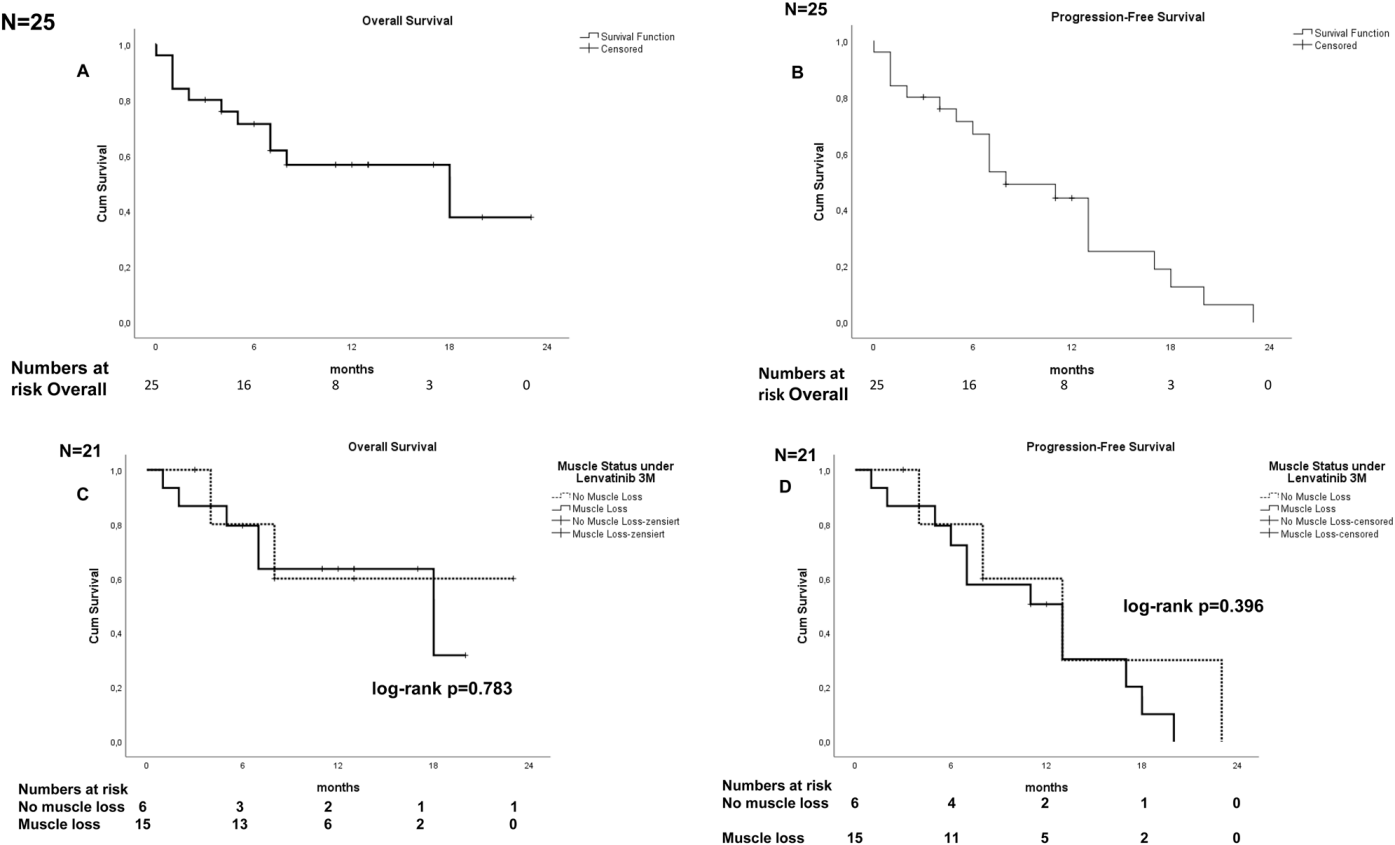
(**A**,**B**) Kaplan–Meier curves for OS (**A**) and PFS (**B**) in all patients with HCC treated with lenvatinib (n = 25). Median OS and median PFS were 18.0 months (95% CI 0.42, 35.58) and 8.0 months (95% CI 3.52, 12.48), respectively. (**C**,**D**) Kaplan–Meier curves for OS (**C**) and PFS (**D**) in sarcopenia patients (n = 15) and non-sarcopenia (n = 6) patients during three months of lenvatinib therapy. Median OS was 18 months in the sarcopenia and in the non-muscle loss group (*p* = 0.783). Median PFS was 13 months in sarcopenia patients and in non-sarcopenia patients, respectively (*p* = 0.396). *OS* overall survival, *PFS* progression free survival.

Efficacy of lenvatinib in our cohort of patients was similar to published data from randomized trials and other retrospective analyses. The estimated median OS and median PFS for all patients (n = 25) were 18.0 months (95% CI 0.42, 35.58) and 8.0 months (95% CI 3.52, 12.48), respectively (Figs. 2A and 2B). There were no complete responses (Table 3), while 16% (4) achieved partial remission (PR) as best response to lenvatinib and 20% (5) stable disease (SD). The objective response rate (ORR) was 16% and the disease control rate (DCR) was 36%.

**Table 3:** Adverse events during lenvatinib therapy.

	Any grades	Grade ≥ 3	Any grades		grade ≥ 3		*p*	*p*
	(All patients, n = 25)	(All patient n = 25)	Non muscle loss during 3 months lenvatinib n = 6*	Muscle loss during 3 months lenvatinib n = 15*	Non muscle loss during 3 months lenvatinib n = 6*	Muscle loss during 3 months lenvatinib n = 15*	Any grade	grade ≥ 3
Total treatment related AEs	25 (100%)	15 (60%)	6 (100%)	15 (100%)	5 (83.3%)	5 (33.3%)	–	0.0635
Fatigue	16 (64%)	2 (8%)	5 (83.3%)	7 (46.7%)	1 (16.7%)	0	0.1778	0.2857
Decreased weight	4 (16%)	1 (4%)	0	4 (26.7%)	0	1 (6.7%)	0.2807	1
Mucositis	8 (32%)	1 (4%)	4 (66.7%)	3 (20%)	1 (16.7%)	0	1	0.2857
Diarrhoea	7 (28%)	2 (8%)	2 (33.3%)	5 (93.3%)	1 (16.7%)	1 (6.7%)	1?	0.500
Hypertension	7 (28%)	0	2 (33.3%)	4 (26.7%)	0	0	1	–
Decreased appetite	7 (28%)	0	2 (33.3%)	3 (20%)	0	0	0.5975	–
Polyneuropathy/tremor	5 (20%)	0	1 (16.7%)	4 (26.7%)	0	0	1	–
Infection	6 (24%)	3 (12%)	2 (33.3%)	2 (13.3%)	1 (16.7%)	1 (6.7%)	0.5439	0.500
Vertigo	4 (16%)	0	1 (16.6%)	2 (13.3%)	0	0	1	–
Pain	6 (24%)	1 (4%)	0	4 (26.7%)	0	0	0.2807	–
Proteinuria	4 (16%)	0	2 (33.3%)	2 (13.3%)	0	0	0.5439	–
Liver funtion disorder Elevated AST/ALT	3 (12%)	2 (8%)	0	2 (13.3%)	0	1 (6.7%)	1	1
Nausea	3 (12%)	0	1 (16.7%)	2 (13.3%)	0	0	1	–
Dyspnoe	2 (8%)	0	0	1 (6.7%)	0	0	1	-–
Neutropenia	2 (8%)	1 (4%)	1 (16.7%)	1 (6.7%)	1 (16.7%)	0	0.500	0.2857
decreased platelet count	2 (8%)	1 (4%)	1 (16.7%)	1 (6.7%)	0	1 (6.7%)	0.500	1
Arterial lung embolie	2 (8%)	2 (8%)	2 (33.3%)	0	2 (33.3%)	0	0.0714	0.0714
Pleura effusion/edema	2 (8%)	0	0	2 (13.3%)	0	0	1	–
Constipation	1 (4%)	0	0	1 (6.7%)	0	0	1	–
Hand-foot syndrome	1 (4%)	0	0	1 (6.7%)	0	0	1	–
Pruritus	1 (4%)	0	0	1 (6.7%)	0	0	1	–
hepatic encephalopathy	1 (4%)	0	0	1 (6.7%)	0	0	1	–
Increased Bilirubin/INR	1 (4%)	1 (4%)	0	1 (6.7%	0	1 (6.7%)	1	1

Numbers are presented as n (%).

The table includes treatment-related adverse events (AEs) of any grades and grade ≥ 3, observed during treatment with lenvatinib.

*Only 21 patients were evaluated, since four patients died before first CT staging.

Regarding efficacy of lenvatinib in patients who developed muscle loss in the first three months of lenvatinib, the muscle loss group showed similar OS of 18.0 months (95% CI 2.12, 33.89) compared to all patients and PFS of 13.0 months (95% CI 7.14, 18.86). Compared to the non-muscle loss group, OS and PFS in patients of the muscle loss group did not differ significantly (*p* = 0.783 and *p* = 0.396, respectively), (Figs. 2C and 2D). ORR was 16.7% in the non-muscle loss group and 20% in the muscle loss group (Table 3) (*p* = 1). Partial response was observed in 1 patient (16.7%) in the non-muscle loss group versus in three patients (20%) in the muscle loss group. DCR was 26.7% in the muscle loss group versus 66.7% in the non-muscle loss group.

### Analysis of factors potentially associated with OS

**Figure 3:**
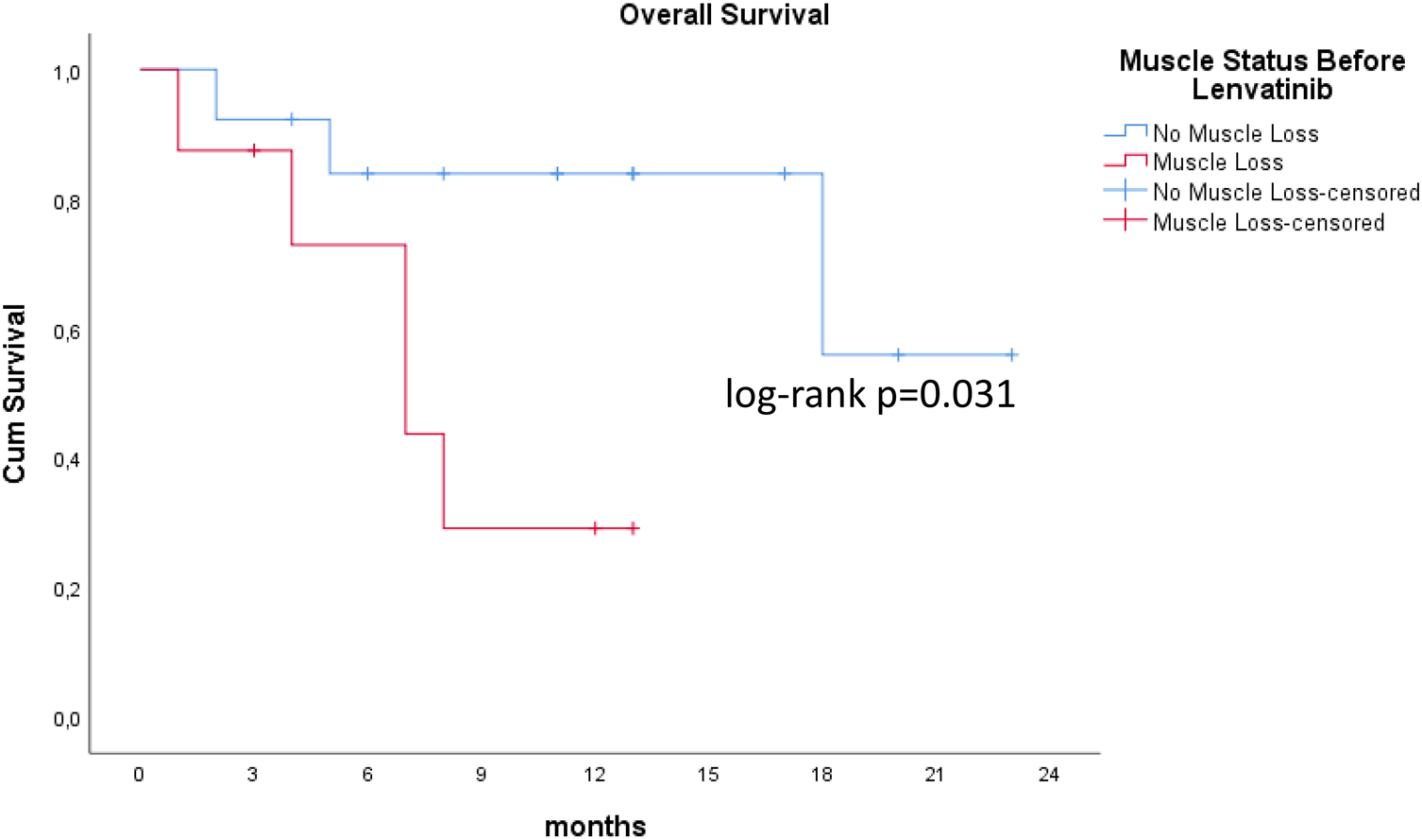
Muscle status of patients before onset of lenvatinib therapy. Median OS was seven months in the muscle loss group and 18 months in non-sarcopenia patients (p = 0.031).

Muscle status before start of lenvatinib therapy was also evaluated. As shown in Table 2, 44% (11) of all patients (n = 25) presented muscle loss at baseline. Patients suffering from muscle loss before starting lenvatinib therapy seem to have shorter survival in terms of OS and PFS than patients without muscle loss (Fig. 3) (*p* = 0.031).

To further clarify the role of muscle loss before and during lenvatinib therapy, univariate analysis, including prognostic parameters, such as age, ALBI, Child–Pugh, ECOG, serum AFP ≥ 200 ng/ml and portal vein infiltration, were included together with muscle loss before and during lenvatinib therapy. As shown in Table 4, in the univariate analysis, Child–Pugh score was identified as predictor of survival (HR 2.47, 95% CI 1.217, 5.012). However, presence of muscle loss before onset of lenvatinib therapy was not found to be a negative predictor of survival. Decreasing SMI during the first three months of lenvatinib therapy was not identified as a poor prognostic factor. Multivariate analysis was not applicable due to small sample size.

**Table 4:** Univariate regression analysis of factors associated with OS.

Parameter	*p*	HR	95% CI
Age	0.137		
ECOG	0.232		
ALBI	0.056		
Child–Turcotte–Pugh score	0.012	2.472	1.217–5.012
Serum AFP	0.461		
Portal vein infiltration	0.095		
Muscle loss before lenva	0.128		
Muscle loss under lenva 3 M	0.675		

n = 21 only patients with 3 months FU.

*ECOG* Eastern Cooperative Oncology Group performance status; *ALBI* albumin-bilirubin score; *AFP* alpha-fetoprotein.

### Toxicity

In Table 5, all adverse effects (AEs) are documented. In all patients, AEs were observed during the observation period as follows: 40% (10), 16% (4), 12% (3) and 4% (1) discontinued lenvatinib therapy because of disease progression (PD), deterioration in liver function, serious adverse events, or intolerance, respectively. Three patients (12%) completed the therapy during two years. The most common adverse effects in all patients were fatigue, decreased weight, mucositis, diarrhea, hypertension, and decreased appetite. In 60% of patients (15), grade ≥ 3 adverse events were documented.

**Table 5:** Trials evaluating the effect of sarcopenia in patients with advanced HCC and treated with lenvatinib.

First author/year	Country	Study desing	N patients	Methodes	Time of muscle assesment	Cut off value	Comparison	Outcome	*p*
Dong et al.^26^	China	Monocentric Retrospective	40	CT scan: L3 SMI	1 month prior to the initiation of lenvatinib	SMI: M: < 42 cm^2^/m^2^ W: < 38 cm^2^/m^2^	sarcopenia patients versus non-sarcopenia patients	OS PFS	0.024 0.044
Hiraoka et al.^21^	Japan	Multicentric Retrospecive	151	CT scan: L3- PSI	1 month before and 4 weeks after starting lenvatinib treatment	SMI: M: 4.24 cm^2^/m^2^ W: 2.50 cm^2^/m^2^	Pre-sarcopenia vs non pre-sarcopenia	OS PFS	< 0.001 0.025
Kotoh et al.^25^	Japan	Monocentric, Retrospective	53	CT scan: L3-SMI HGS: GS dynamometer	Before receiving lenvatinib	SMI: M: < 42 cm^2^/m^2^ W: < 38 cm^2^/m^2^ HGS M: < 26 kg W: < 18 kg	HGS: Low versus High Muscle depletion versus Non-Muscle depletion Sarcopenia versus non- Sarcopenia	OS TTF	0.036
Endo et al.^23^	Japan	Monocentric Retrospective	63	CT scan: L3-SMI GS: measurement complies with the established guideline (JSH)	Before receiving lenvatinib	GS: M: < 26 kg W: < 18 kg SMI: M: < 42 cm^2^/m^2^ W: < 38 cm^2^/m^2^	GS: Normal versus decreased SMI: Normal SMI vs decreased SMI,	OS PFS PPS	0.9
Uojima et al.^24^	Japan	Multicentric Retrospective	100	CT scan: L3-SMI	before treatment initiation	SMI: M: < 42 cm^2^/m^2^ W: < 38 cm^2^/m^2^:	SMI: low vs high	TTF OS	0.010 0.021
Rinninella et al.^22^	Italy	Two case Reports Retrospective	2	CT scan: L3- SMA	At baseline and at 24 months after lenvatinib skeletal muscle area treatment	–	SMA, Baseline versus 24 months after the start of lenvatinib therapy	OS PFS	–

*SM* skeletal muscle; *SMI* skeletal muscle index, calculated as skeletal muscle mass divided by height squared (cm^2^/m^2^); *HGS* handgrip strength; *L3* third lumbar vertebra level; *OS* overall survival; *MST* median survival time; *n.s.* non significant; *PFS* progression free survival; *PPS* post-progression survival; *SMA* skeletal mass area; *PSI* psoas muscle area index (muscle area at level of middle of third lumbar vertebra (cm^2^)/height (m^2^); *TTF* time to treatment failure.

Interestingly, patients developing muscle loss had fewer severe adverse effects. However, reduced rate of side effects was not significantly different compared to the patients without muscle loss (*p* = 0.06).

## Discussion

In this study, we analyzed the effect of lenvatinib on muscle loss development in a non-selected real-life cohort of patients with advanced HCC. The majority of the patients significantly developed progressive muscle loss during the first three months of lenvatinib therapy. Outcome of all patients in terms of OS, PFS and ORR was similar to the published data in the pivotal prospective randomized phase III trial^7^. Despite muscle loss, patients benefitted from the therapy with lenvatinib since outcomes and toxicity were similar to the outcome of patients without muscle loss.

HCC occurs mostly as a consequence of liver cirrhosis and chronic pre-existing liver diseases. As a result of these comorbidities, decreased physical activity and nutritional deficiencies are very common in these patients, causing loss in weight and muscle volume. Especially sarcopenia, which is defined as a significant loss of skeletal muscle mass, quality and/or function seemed to have a relevant prognostic role in these patients. In recent works, we and others elucidated the role of sarcopenia in the outcome of patients with liver cirrhosis^11,12,16,17^.

Shachar et al.^14^ described in a meta-analysis that up to 74% of patients with advanced tumors were sarcopenic and that sarcopenia has a significant impact on cancer outcomes including OS and PFS. Other authors report from significant interactions between treatment and Low skeletal muscle mass (LSMM) in oncology. Some machnisms are involved in this process. Patients with extrahepatic cholangiocarcinoma and sarcopenia showed a decreased average count of CD8 + T cells compared to those without sarcopenia^28^. Skeletal muscle cells present antigens via major histocompatibility complexes I and II and influencing T cell function^29^. Additionally, skeletal muscles produce cytokines (myokines) with immune effects such as interleukin(Il)-15 which stimulates the proliferation and activation of natural killer cells and CD8 + T cells^30,31^.

Sarcopenia is not a rare finding in HCC patients, with a high prevalence of LSMM. A significant correlation was observed between the presence of LSMM and decreased overall survival in patients with HCC in both univariable and multivariable analyses^32^. Guo et al. reported in a meta-analysis that sarcopenia is linked to significantly reduced overall survival (OS), an increased risk of tumor recurrence, poorer tumor response, and more drug-related adverse events in patients with HCC. The presence of cirrhosis and Child Pugh class B raises the mortality risk associated with sarcopenia^33^. The relationship between skeletal muscle mass loss and survival was analysed in a meta-analysis. The analyses showed that sarcopenia was associated with an increase in overall mortality and a higher risk of tumour recurrence^34^.

Moreover, several tyrosine kinase inhibitors (TKI), including sorafenib and lenvatinib, may increase sarcopenia. In patients with advanced HCC and treated with sorafenib, sarcopenia seems to be a prognostic factor for mortality and an independent factor for early dose-limiting sorafenib toxicities^20,34^.

Lenvatinib has been approved since 2018 for first-line therapy of advanced HCC based on the results of the phase III REFLECT trial. Although sarcopenia as a possible prognostic parameter before and during therapy with lenvatinib was not analyzed in the REFLECT trial^7^, the high frequency of patients with loss of appetite and weight reported during the therapy with lenvatinib, suggests a possible role in sarcopenia development which can influence on the outcome of patients^8,20^.

Regarding the role of sarcopenia in patients with advanced HCC and treated with lenvatinib, only limited data, mostly from Japan, have been published to date. Confirming our hypothesis, in our cohort of patients from Germany, we detected a significant decrease of muscle loss (6.5% ± 3.3%; *p* = 0.035) in the first three months of lenvatinib therapy in the majority of patients (60%). Moreover, this effect was observed not only in patients with previous muscle loss but also in patients without previous muscle loss. It has been described that several multikinase inhibitors, mainly targeting vascular endothelial growth factor (VEGFR/VEGF) signal cascade, are suspected of inducing muscle mass loss. VEGF seems to promote the proliferation of myogenic fibers. Thus, the effect of TKI could inhibit muscle growth due to this mechanism, exacerbating the muscle mass loss and inducing sarcopenia. Lenvatinib may inhibit tumor cell proliferation by downstream downregulation of PI3K, AKT and mTOR. Further mechanisms, such as increased systemic inflammatory response and decreased protein synthesis due to chronic liver disease and cancer, are also involved in the aggravation of muscle mass loss in these patients^35^.

Despite the significant muscle loss in our cohort of unselected patients, we observed a benefit in terms of mOS of 18.00 months (95% CI 0.42, 35.58), PFS of 8.00 months (95% CI 3.52, 12.48) and ORR of 16% due to systemic therapy with lenvatinib. In the REFLECT study, lenvatinib showed an OS of 13.6 months (95% CI 12.1, 14.9). The achieved ORR was 24.1% and PFS was 7.4 months (95% CI 6.9–8.8 months). Regarding toxicity, grade ≥ 3 adverse events occurred in 60.0% of patients in our cohort during lenvatinib therapy, which is higher than the results of the REFLECT trial, where serious adverse events were observed in 43.1% of patients. Similar to the REFLECT trial, the most common adverse events found in our cohort were decreased weight in four patients (16%), mucositis in eight (32%), hypertension in seven (28%), diarrhea in seven (28%) and decreased appetite in seven patients (28%). Analyzing the base line characteristics of patients included in the REFLECT study, we found several differences compared to the characteristics of our cohort of patients. For instance, in the REFLECT trial, patients had BCLC stage B or C, Child–Pugh class A cirrhosis and ECOG score of 0 or 1. Additionally, patients with 50% or more liver occupation and/or bile duct or main portal vein invasion were excluded. Our real-life cohort of patients included 20% with Child Pugh B liver cirrhosis, 24% had ECOG ≥ 2 and 48% main infiltration of the portal vein, which were not included in the REFLECT study. Moreover, 100% of our patients had BCLC C, while only 78.2% of patients in the REFLECT trial were BCLC C. Finally, 20% of our patients received lenvatinib after at least one prior systemic therapy. Thus, our findings showed that lenvatinib was beneficial and safe in a European unselected cohort of patients with advanced HCC in daily clinical practice, confirming the data from other real-life cohorts of patients from Asia published to date^36–39^.

Most importantly, in this study, treatment with lenvatinib in patients developing muscle loss was also found to be beneficial in terms of OS and PFS. The mOS of the patients with muscle loss was 18.00 months (95% CI 2.12, 33.89) and the PFS 13.0 months (95% CI 7.14, 18.86). This was not significantly different compared to the patients without muscle loss development (*p* = 0.783 and *p* = 0.396, respectively). ORR was comparable between the two groups. Partial response was observed in 16.7% (1) in the non-muscle loss group versus 20% (3) in the muscle loss group. In the non-muscle loss group, 50% (3) achieved SD. Regarding toxicity, there was no significant increase in side effects in patients who developed muscle loss during lenvatinib therapy. Indeed, patients developing muscle loss had fewer severe adverse effects, including less worsening of liver function.

We also analyzed the impact of previous muscle loss on survival in patients treated with lenvatinib and found that suffering from previous sarcopenia before starting lenvatinib therapy (44%) showed that in these patients, survival in terms of OS and PFS was shorter than in patients without previous sarcopenia. However, in the univariate analysis, presence of muscle loss at onset of lenvatinib therapy and the development of muscle loss were not found to be negative predictors of survival.

Comparing our study to data published to date, ours is one of the first analyses on skeletal muscle evolution during lenvatinib therapy in patients with advanced HCC from a European cohort of patients (Table 1).

In our study, CT imaging was selected to quantify muscle mass. This non-invasive method allows correlation with the whole-body mass. Moreover, we and others have already shown that this muscle mass measurement is a strong predictor for mortality in patients with cirrhosis and HCC^16,17^. However, some of the studies with HCC patients treated with lenvatinib published to date (Table 1) showed the effect of handgrip strength rather than skeletal muscle mass as a negative predictor of survival.

Since all published studies to date included only patients from Asia, mostly from Japan, and quantitative and qualitative assessments of sarcopenia with different cut-off values among the published studies were presented, further direct comparisons among the studies are difficult. Nutritional habits and body composition may differ considerably between Japan and Europe with different impact on the effects of muscle mass during therapy with lenvatinib. To date, there are limited comparable data available including patients from outside Asia.

More data from other cohorts of patients are warranted to clarify the role of sarcopenia in the treatment of patients with advanced HCC. Moreover, preventive and therapeutic interventions to avoid or to reduce sarcopenia, as already indicated^20^, should be included and investigated in clinical studies in more detail.

The limitations of the present study include the relatively small number of patients and its retrospective design with patients of a single institution. Therefore, lack of significance between the patients without development of muscle loss and the patients with development of muscle loss could be related to the low number of patients. Moreover, four patients died before assessment of skeletal muscle mass three months after onset of lenvatinib therapy. Thus, sample selection may be biased. We evaluated only a quantitative parameter of skeletal muscle mass, but no quality parameters of the muscle mass, such as grip strength.

The strengths of this trial are the inclusion of a European cohort of patients with different clinical body and nutritional features compared to patients from Japan and the assessment of sarcopenia not only at baseline but also over the course of therapy with lenvatinib.

In summary, our study in an unselected European cohort of patients with advanced HCC and treated with lenvatinib showed similar outcome in terms of OS, PFS and ORR to published data in the pivotal prospective randomized phase III trial (REFLECT). Three months after starting lenvatinib therapy, most patients experienced a significant loss of skeletal muscle mass. However, despite of developing muscle loss, patients benefitted from the therapy with lenvatinib. No significant difference in OS and PFS between the group of patients developing muscle loss and the non-muscle loss group was found.

Further studies are necessary to elucidate the role of muscle loss in patients with advanced HCC treated with lenvatinib. Since several studies showed a negative effect of sarcopenia on OS and tolerability to TKI in cancer patients, including HCC, sarcopenia assessment before and during lenvatinib should be taken into account and, if applicable, nutritional and sports measures should be started early on to prevent or improve sarcopenia both in clinical trials and in everyday practice in order to improve prognosis.

## Patients and methods

### Patient characteristics

In total, 25 patients with advanced HCC (BCLC C) treated with lenvatinib between June 2018 and March 2021 at the University Hospital of Bonn, Germany, were included in this study. Diagnosis of HCC was confirmed by histological or radiological validation according to current European guidelines^5,6^. Individual patient treatment was determined after the cases had been discussed in weekly interdisciplinary tumor conferences by representatives from all departments of oncological gastroenterology. Patients were considered for systemic therapy with lenvatinib when all other curative options had been ruled out. Systemic therapy with lenvatinib was offered if performance status and hepatic and renal function were considered sufficient. At the time of therapy decision, only sorafenib or lenvatinib were approved as first-line therapies for advanced HCC. Patients with poor liver function (Child–Pugh B, 8 points) were mostly offered sorafenib or best supportive care. Lenvatinib was also offered as last-line therapy (second-fourth line) as off label use, when all available palliative standard therapies had been ruled out. Baseline characteristics were recorded prior to lenvatinib therapy. Baseline and therapy characteristics before and during lenvatinib therapy were collected by reviewing medical records and images. Relevant comorbidities and all previous therapies of HCC as well as all therapies after lenvatinib are documented in Table 2.

### Treatment regimen with lenvatinib

#### Assessment of response and toxicity

Therapy with lenvatinib was scheduled based on the standard dose used in the REFLECT trial: 8 mg for patients weighing < 60 kg and 12 mg for patients weighing ≥ 60 kg orally once a day. Patients with apparent risk factors received a reduced initial dose of lenvatinib (4 or 8 mg) in order to avoid increase of toxicity for 5–7 days.

All patients received lenvatinib continuously until toxicity or progression of tumor disease. Staging examinations by thoracic and abdominal CT and/or additional liver magnetic resonance tomography (MR) were carried out every 6–12 weeks. Response was classified according to modified Response Evaluation Criteria in Solid Tumors (mRECIST) and/or RECIST 1.1. Objective response rate (ORR) was defined as complete response (CR) and partial response (PR), and disease control rate (DCR) was defined as CR, PR, and stable disease (SD). Side effects during the therapy with lenvatinib were recorded according to the common terminology criteria for adverse events (CTCAE, version 5.0) (Table 5).

### Assessment of muscle mass

Muscle measurements were carried out based on the CT images of the patients at different time points: at the initial diagnosis of HCC, at the start of therapy with lenvatinib and at every 12 weeks in each staging.

Typical imaging parameters included: slice thickness 1 or 2 mm, tube current (exposure time product) 100 mAs, tube voltage 120 kVp.

Skeletal muscle area at the intervertebral disc space level between the third and fourth lumbar vertebra were used for estimation of skeletal muscle mass in this study. Lean muscle tissues were identified by ranges of high attenuation (30–100 HU). Any decrease in skeletal muscle area in 3 months was defined as muscle loss.

The images were manually preselected and developed using a method described in the literature, which has been expanded into a fully automatic and high-precision segmentation tool for determining body composition based on abdominal CT scans with the open-source Convolutional Neural Network (CNN) DeepMedic (Fig. S1)^40,41^.

All images were critically reviewed by a body composition analysis expert (S.N) or a certified radiologist (J.L.) and, if necessary, corrected manually.

### Study design

This is a retrospective observational analysis of patients at the University Hospital of Bonn, Germany. Included in this study were 25 patients with unresectable advanced HCC who were treated with lenvatinib between June 2018 and March 2021. Baseline parameters (Table 2) were recorded prior to therapy. Patients were followed until death or March 2021. When lost to follow-up, patients were censored at date of last visit. The primary endpoint was overall survival (OS) and secondary endpoints included progression-free survival (PFS), objective response rate (ORR), disease control rate (DCR), toxicity assessment for all patients and for patients losing muscle during the first three months of lenvatinib therapy (muscle loss group) compared to the patients without loss of muscle (group without muscle loss).

### Statistical analysis

Differences in continuous variables, expressed as medians and first and third quartiles were assessed using non-parametric Mann–Whitney test. Categorical variables, expressed as absolute frequencies and percentages, were compared using Monte Carlo Chi^2^ homogeneity test based on discrete uniform distribution or Fisher’s exact tests. Survival curves were constructed using Kaplan–Meier diagrams and compared using a log-rank test. Univariate analysis was performed using Cox regression models. OS and PFS were expressed as median in months, with 95% confidence interval (CI). Statistical significance was defined as a *p* value ≤ 0.05. All data were analyzed using SPSS (version 24; IBM, Armonk, NY, USA).

### Ethic statement

All methods were carried out in accordance with relevant guidelines and regulations. This retrospective study was approved by the Ethics Committee of the Medical Faculty of the University of Bonn (No. 341/17).Written informed consent wasobtained from all patients.

## Supplementary Information

Supplementary Information.

## Data Availability

The data generated during this study are included in this article.
