# Supplementary material for: The impact of lenvatinib on sarcopenia in patients with advanced unresectable hepatocellular carcinoma
Source: Sci Rep. 2024 Sep 27;14:22146. doi: 10.1038/s41598-024-66766-8 (PMC11437060; doi:10.1038/s41598-024-66766-8)
Supplement: Supplementary file 1 — Supplementary Information. [file 41598_2024_66766_MOESM1_ESM.pptx]

## Slide 1
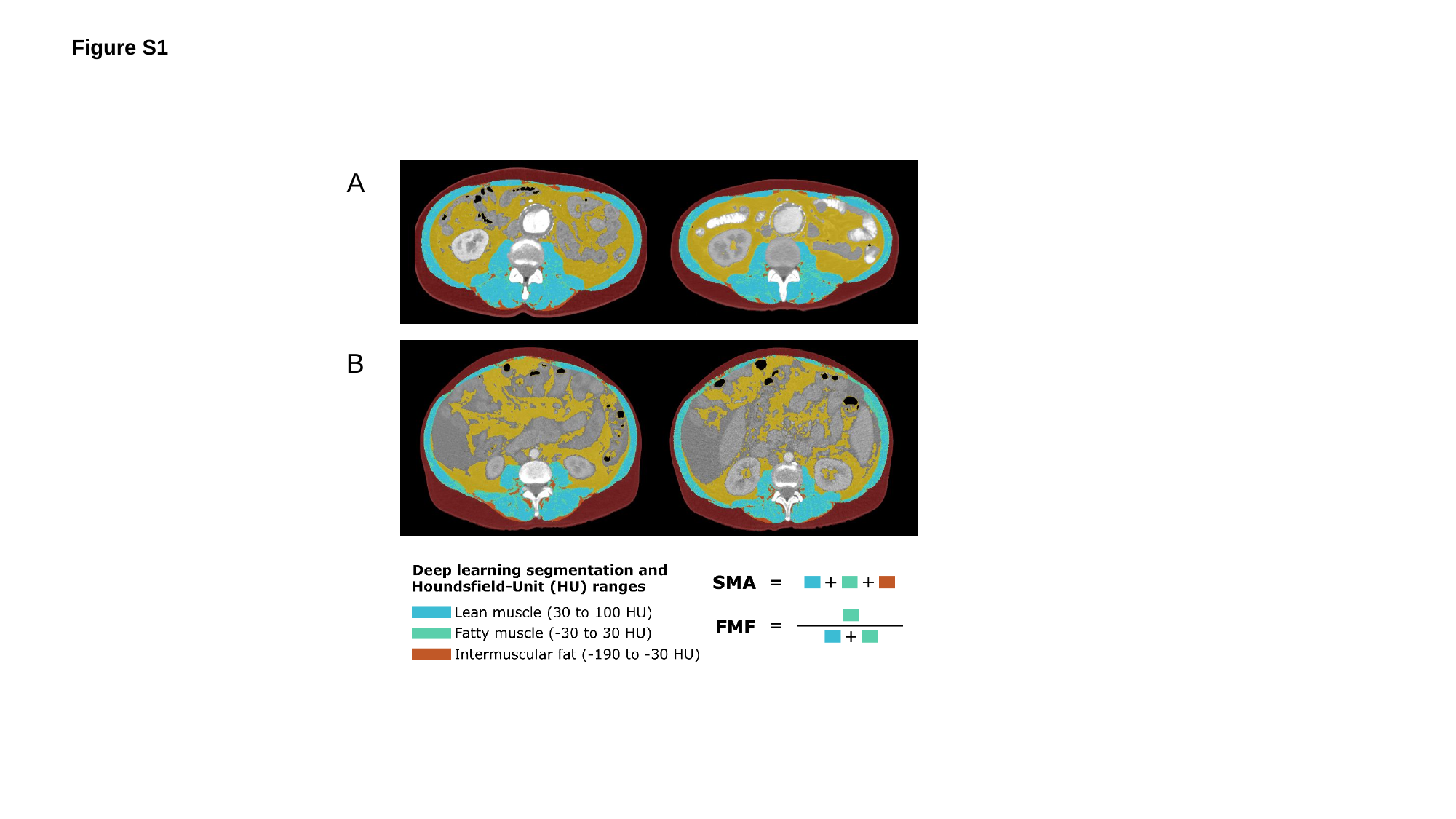

Figure S1
A
B

## Slide 2
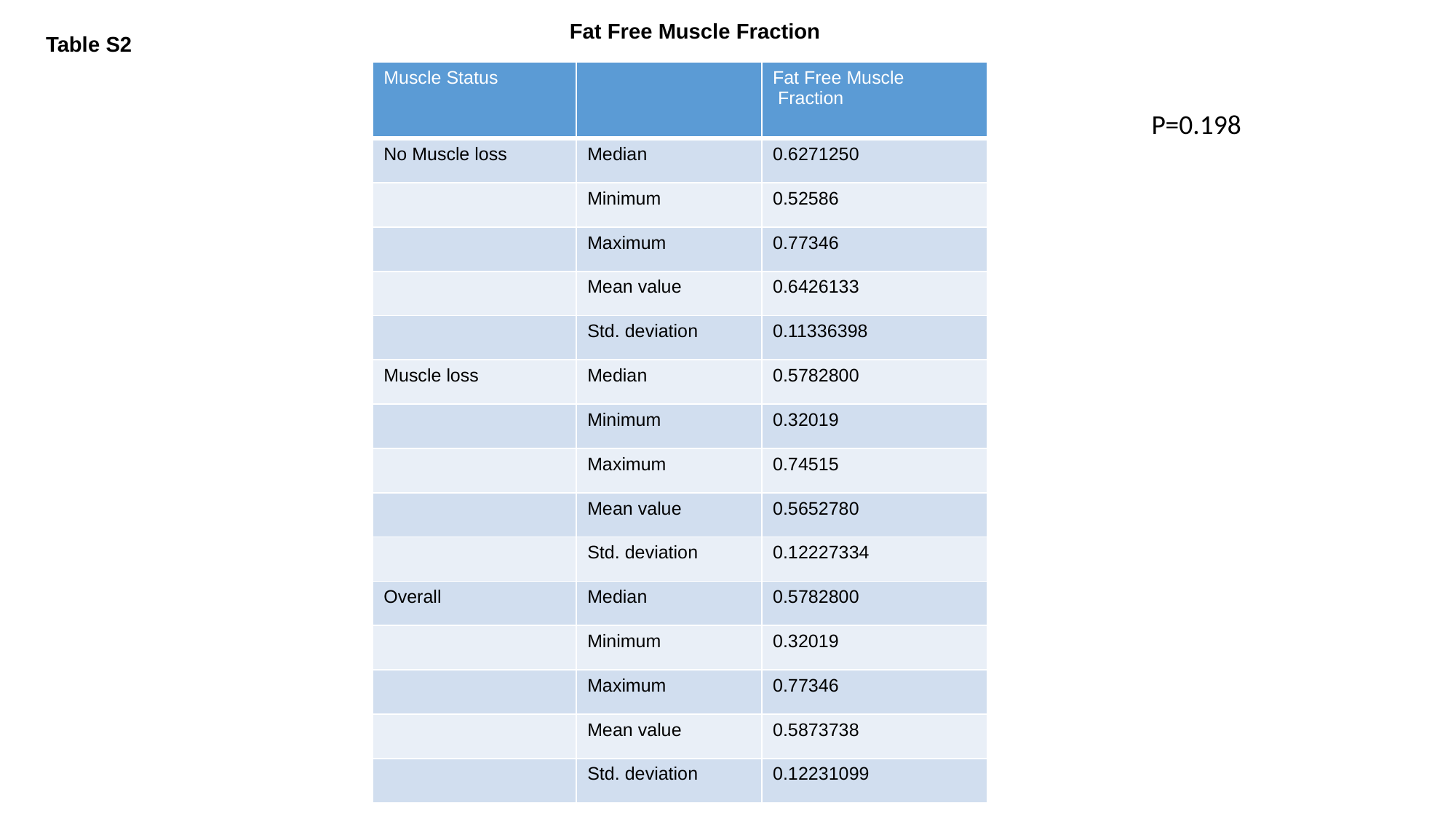

Fat Free Muscle Fraction
Table S2
| Muscle Status | | Fat Free Muscle Fraction |
| --- | --- | --- |
| No Muscle loss | Median | 0.6271250 |
| | Minimum | 0.52586 |
| | Maximum | 0.77346 |
| | Mean value | 0.6426133 |
| | Std. deviation | 0.11336398 |
| Muscle loss | Median | 0.5782800 |
| | Minimum | 0.32019 |
| | Maximum | 0.74515 |
| | Mean value | 0.5652780 |
| | Std. deviation | 0.12227334 |
| Overall | Median | 0.5782800 |
| | Minimum | 0.32019 |
| | Maximum | 0.77346 |
| | Mean value | 0.5873738 |
| | Std. deviation | 0.12231099 |
P=0.198
